# Green Leafy Vegetables (GLVs) as Nutritional and Preventive Agents Supporting Metabolism

**DOI:** 10.3390/metabo15080502

**Published:** 2025-07-28

**Authors:** Renata Nurzyńska-Wierdak

**Affiliations:** Department of Vegetable and Herb Crops, Faculty of Horticulture and Landscape Architecture, University of Life Sciences in Lublin, Doświadczalna 50a, 20-280 Lublin, Poland; renata.nurzynska@up.lublin.pl

**Keywords:** metabolic diseases, prevention, healthy diet, vegetable/vegetable products, bioactive substances

## Abstract

Metabolic syndrome (MetS) is defined as a group of metabolic defects that include hypertension, insulin resistance, visceral obesity, fatty liver disease, and atherosclerotic cardiovascular disease (CVD). The first step in controlling the progression of MetS is lifestyle changes, including dietary modification. Regular consumption of fruits, vegetables, whole grains and other plant foods negatively correlates with the risk of developing chronic diseases. Green leafy vegetables (GLVs) are a key element of healthy eating habits and an important source of vitamins C and E, carotenoids—mainly β-carotene and lutein—and minerals. This review discusses and summarizes the current knowledge on the health benefits of consuming GLVs in the prevention and treatment of MetS to provide a compendium for other researchers investigating new natural products.

## 1. Introduction

Humans are exposed to an increasing number of environmental hazards, some of which have been identified as important risk factors for metabolic diseases such as diabetes and obesity. These diseases typically occur when key metabolic and signalling pathways that pollutants may affect are disrupted, along with genetic and lifestyle factors [1]. Metabolic syndrome (MetS) is defined as a group of metabolic defects, including hypertension, insulin resistance, visceral obesity, hepatic steatosis and atherosclerotic cardiovascular disease (CVD). The first step in controlling the progression of MetS is lifestyle changes. The available pharmacotherapy and associated comorbidities require the continuous use of multiple medications, creating the risk of polypharmacy, dangerous for everyone, but especially for older people with a chronic disease burden. Recently, special attention has been given to natural foods as potential preventive and therapeutic agents in MetS [2]. Vegetables are a rich and still untapped source of bioactive compounds, attractive for their health-promoting properties and as raw material for developing pharmaceutical and food products. This review discusses and summarises the existing knowledge on the health benefits of consuming green leafy vegetables (GLVs) in the prevention and treatment of MetS to provide a compendium for other researchers investigating alternative health approaches and new natural products. This review aims to provide an overview of the main biological activities of GLVs, particularly metabolic support activities, and to discuss the nutritional, health-promoting and dietary benefits of GLVs using selected examples.

## 2. Metabolic Diseases—A Health and Social Problem

The importance of MetS lies in its associated risk of CVD and type 2 diabetes mellitus (T2DM), as well as other harmful conditions such as non-alcoholic fatty liver disease. A healthy lifestyle is key to preventing or delaying the onset of MetS in susceptible individuals and preventing CVD and T2DM in those with existing MetS. Weight reduction through an energy-restricted diet and increased energy expenditure through physical activity contribute to the prevention and treatment of MetS. This dietary regimen considers several bioactive substances in fruit and vegetables, including antioxidant compounds [3]. MetS and its associated conditions are a major global societal problem today. The prevalence of MetS in obese children is documented to be around 30%. As the components of MetS show stability from childhood to adulthood, children who meet the diagnostic criteria for MetS may remain at high risk. The severity of MetS in childhood has been linked to future risk of T2DM and CVD. Implementing healthy lifestyle interventions in early childhood may be preventative against the future development of MetS and its complications. The primary goal of MetS treatment is to mitigate all metabolic risk factors through effective lifestyle changes. One of the key elements of prevention in this regard is an adequate diet enriched with protective and metabolism-supporting foods. The recent epidemics of metabolic diseases, obesity, T2DM, hepatic lipid disorders, and MetS are primarily attributed to genetic background and changes in diet, exercise and ageing [4]. When discussing dietary changes, attention should be paid to the increased consumption of vegetables, including GLVs. As a low-calorie food, GLVs are ideal sources of valuable nutrients in the daily diet. Cook et al. [5] showed that consuming certain types of vegetables by obese young Latinos is associated with positive metabolic outcomes, including reductions in visceral and liver fat and T2DM risk factors, even when consumed in small amounts. Consumption of dark green and intense orange/yellow vegetables correlated positively with insulin sensitivity (r = 0.19, *p* = 0.03). Consumers of these foods (mean intake = 0.3 ± 0.4 portions/day, n = 107), compared with non-consumers (n = 68), had 31% increased insulin sensitivity (1.6 ± 1.6 vs. 2.1 ± 1.3 × 10^−4^-min^−1^-μU^−1^-mL^−1^, *p* = 0.03) and 17% less visceral fat (2.3 ± 0.9 vs. 1.9 ± 0.7 L, *p* = 0.01). Consuming sufficient GLVs may be beneficial in reducing the risk of MetS in children and adolescents [6]. GLVs such as spinach, arugula, and Swiss chard have been shown to reduce MetS symptoms, possibly because they contain dietary fiber, nitrates and flavonoids, among others [7].

## 3. Vegetables in the Human Diet

**Figure 1:**
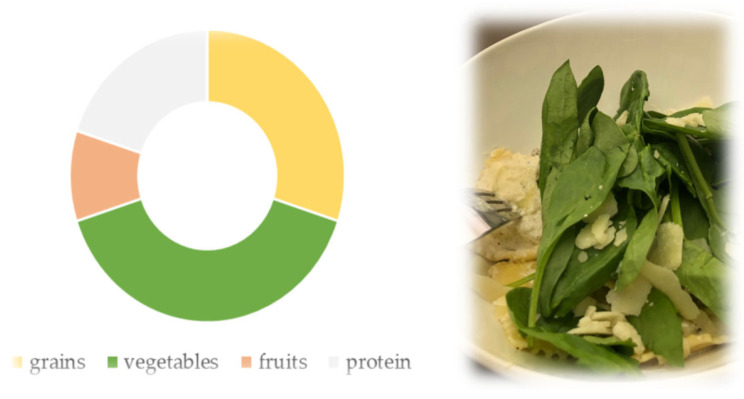
Visual representation of MyPlate divided into four sections: grains (30%), vegetables (40%), fruits (10%), and protein supplemented with dairy: a glass of milk or a cup of yogurt (20%). Example combination of GLVs and protein.

CVDs are the most common non-communicable diseases, leading to death and disability worldwide. They represent a significant health challenge as they are responsible for more than 20 million deaths worldwide. Unhealthy lifestyles (limited physical activity, poor diet, stress) cause vascular dysfunction, followed by hypercholesterolemia, hypertension, and T2DM. Lifestyle interventions focused on lowering blood glucose, lipids, and blood pressure have shown efficacy in mitigating the risk of developing CVD risk factors. The USDA’s MyPlate guidelines promoting healthy eating recommend a 2:1:1 plate ratio, with half allocated to fruits and vegetables and half to cereals and protein [8,9]. Kaewpradup et al. [10] analyzed the effects of dietary enrichment with eight GLVs from the Asteraceae and Brassicaceae families at 0.5, 1.0, and 1.5 times the MyPlate recommendation (Figure 1). Significant total polyphenol content of 5.77–9.46 mg gallic acid equivalent (GAE)/g extract was found, and nitrate accumulation was higher in Asteraceae vegetables (590.90–1155.04 mg NO_3_-NE/g extract) than in Brassicaceae (244.96–726.20 mg NO_3_-NE/g extract). Inclusion of ≥1 serving of all GLVs significantly reduced fast and slow digestible starch fractions, while undigested starch increased significantly, resulting in delayed glucose release. There was also a significant increase in antioxidant activity (AA) with ≥1 serving and reduced free fatty acid concentrations with larger portions of vegetables. Post-digestion nitrate concentrations ranged from 127.3 to 188.5 μg NO_3_-N/mL, positively correlating with GLVs portion size. These effects were dose- and vegetable-species-dependent. The authors suggest including GLVs at or above the MyPlate recommendation may protect CV system health.

**Figure 2:**
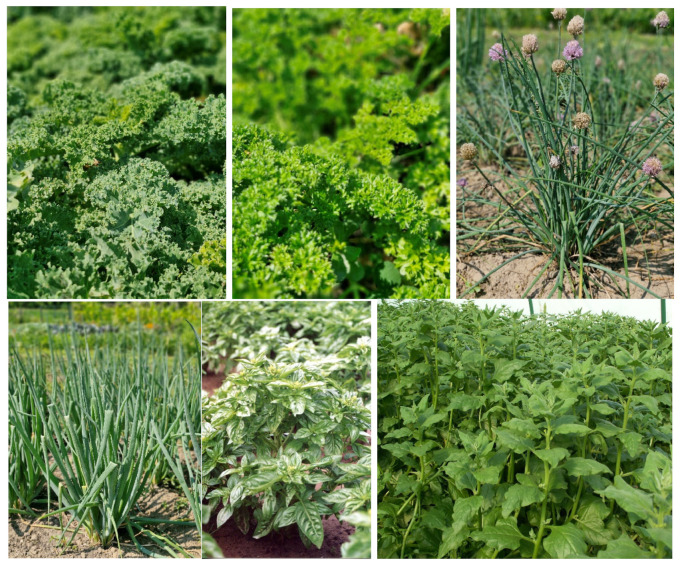
Examples of plants from the green leafy vegetables (GLV) group. From top/left: *Brassica oleracea* L. var. *sabellica* L., *Petroselinum crispum* (Mill.) Fuss. subsp. *crispum*, *Allium schoenoprasum* L., *Allium cepa* L., *Ocimum basilicum* L., and *Tetragonia tetragonioides* (Pallas) Kuntze.

Regular consumption of fruit, vegetables, whole grains, and other plant products is negatively correlated with the risk of developing chronic diseases. Vegetables are essential for health as they provide the body with vitamins, dietary fiber, and minerals. They are a rich source of vitamin C, carotene (provitamin A), vitamin E, several B vitamins, polyphenols and minerals, mainly calcium, potassium, magnesium, and iron. They are among the products that deacidify the body. A direct link exists between consuming fruit and vegetables and their disease-preventing effects. According to analyses based on 95 studies, consuming 200 g of fruit and vegetables per day can reduce the risk of diseases by 13%, while with an intake of 800 g per day, the risk reduction reaches almost 30% [11,12]. There are approximately 10,000 plant species used as vegetables worldwide. Their classification can be based on common morphological characteristics (roots, stems, fruits, leaves, etc.), use, or nutritional/health benefits. GLVs are a unique group of vegetable plants that are extremely valuable and popular with consumers (Figure 2). These vegetables are a key part of healthy eating habits and an important source of vitamins C and E, carotenoids, mainly β-carotene and lutein, and minerals. The GLVs group classifies different species of plants with edible leaves gathered in loose leaf rosettes or compact leaf heads. In a guide prepared by the Turkish Ministry of Health, GLVs are classified as green Mediterranean/salad vegetables such as spinach, Swiss chard, quince, blackcurrant, grape leaves, curly lettuce, spinach, purslane, parsley, cress, rocket, mint, sorrel, radish, dill, radicchio, and curly chicory [13]. Due to their global consumption and economic importance, lettuce, spinach, and chicory are the main GLVs. Other species, such as arugula, mizuna, and lamb’s lettuce, are gaining importance due to their use in salad mixes, which are of increasing interest to consumers [14]. A special feature of GLVs is their unique chemical composition and properties. These plants contain cellulose, hemicellulose, and pectic substances, which give them texture and firmness, as well as dietary fiber, protein, minerals, vitamins, and other bioactive components. In addition to enriching the menu, they contribute nutrients and health-promoting ingredients usually lacking in the daily diet. GLVs also exhibit protective properties and are useful for maintaining health and preventing diseases [15,16]. GLVs require careful handling during harvesting, storage, and processing to preserve their quality and biological value. Particular attention is paid to proper washing, cooling, and packaging to eliminate damage and microbial growth. GLVs are mostly consumed raw, thus retaining all their valuable nutrients. Some species can be stored and processed (boiled, fried, pickled, marinated, etc.). In a diet dominated by starchy staples (cereals), GLVs significantly contribute to food security and dietary variety [17].

## 4. Some Helpful GLVs Components in Metabolic Disease

The nutrient composition of vegetables is highly complex and variable. The level of metabolites is strongly influenced by genetic and environmental factors, as well as transport, storage and/or processing conditions. Growth factors such as light, temperature, humidity, soil, type of agrotechnical treatment, damage by microorganisms and insects, UV stress, and environmental pollutants alter the composition of plant metabolites [18]. Vegetables grown in peri-urban areas are exposed to higher concentrations of organic micropollutants and trace elements than those grown in rural areas [19]. Using surface water (into which wastewater from wastewater treatment plants flows) for crop irrigation can contaminate crops due to pharmaceuticals, trace metals and metalloids, posing health risks [20]. However, with good agricultural practices and crop recommendations, good-quality products with high nutritional and health-promoting qualities can be offered to consumers. A high intake of GLVs can meet the nutrient requirements for proper growth, thus providing adequate protection against diseases resulting from malnutrition [15]. GLVs such as spinach, lettuce, chard, purslane, chicory, and others are important sources of minerals (iron and calcium), vitamins (A, C, and riboflavin), and fiber. The distribution of nutrients in the edible parts of GLVs is not uniform. Young, fresh leaves contain more vitamin C than mature plants. The green outer leaves of lettuce and cabbage are richer in vitamins, calcium and iron than the white inner leaves. Thinner and greener leaves are more nutritious and usually have fewer calories. Dark green leafy vegetables are an important source of vitamins C and K, folate, β-carotene, lutein + zeaxanthin, flavones, and contribute iron, copper, manganese, vitamin B_6_, phytosterols, α-carotene and flavonols. Other leafy vegetables are identified as important sources of vitamins C and K and anthocyanidins, as well as providing phytosterols, manganese, vitamin B_6_, folate, β-carotene, lutein + zeaxanthin [13]. In addition to GLVs, which are well known in cultivation and use, the possibility of similarly using other plants has also been reported [21], including wildflower species [22]. Saini et al. [23] compared the content and composition of bioactive compounds of lettuce and spinach with new GLVs, moringa (*Moringa oleifera* Lam., Moringaceae) and fenugreek (*Trigonella foenum graecum* L., Fabaceae). The predominant carotenoid of the GLVs studied was found to be (all-E)-lutein, ranging from 31.3 (green/red lettuce) to 45.3% (fenugreek) of the total carotenoids, followed by (all-E)-violaxanthin and (all-E)-β-carotene. (all-E)-β-carotene, a provitamin A carotenoid, was the second most dominant carotenoid of *M. oleifera*, accounting for 109.2 μg/g fresh weight (FW). The plant also had the highest levels of total carotenoids (473.3 μg/g FW), α-tocopherol (83.7 μg/g FW) and total phytosterols (206.4 μg/g FW). The authors suggest that *M. oleifera* leaves can serve as an affordable source of nutrients in the diet.

### 4.1. Carotenoids in the Prevention and Treatment of Metabolic Disorders

One of the leading causes of chronic diseases is oxidative stress, caused by the presence of highly active oxygen species, so-called free radicals. The formation of free oxygen radicals is inevitable, but it is possible to protect against them with a diet rich in natural antioxidants. Carotenoids show strong antioxidant activity, reducing the risk of developing CVS, T2DM, and some malignancies. The high biological activity of carotenoids and their derivatives supports their use as alternative MetS therapeutics, as they reduce serum triglyceride levels, promote insulin response, inhibit adipogenesis and reduce angiotensin-converting enzyme activity [24,25]. In C57BL/6J obese mouse models, lutein treatment was shown to reduce epididymal and abdominal fat mass, serum cholesterol, low-density lipoprotein cholesterol (LDC-C), hepatic triglycerides and cholesterol, and blood glucose levels. Lutein supplementation may, therefore, control diet-induced obesity and its associated complications [26]. Carotenoids are particularly sensitive to pH, light exposure, and temperature. Their content can change with food processing, cooking, or storage methods, given the remarkable biological activity of carotenoids and their susceptibility to degradation due to environmental and physiological factors. Introducing high-carotenoid products into the diet, including GLVs (Table 1), is advisable.

GLVs are an important source of carotenoids, mainly β-carotene and lutein. The presence of lutein in the diet is significant; this compound reduces the occurrence of many conditions: macular degeneration, cataracts, coronary heart disease, stroke, oesophageal cancer, non-Hodgkin’s lymphoma, metabolic syndrome, and amyotrophic lateral sclerosis. Consumption of 100 g of GLVs per day is associated with a reduced risk of coronary heart disease and stroke mortality by approximately 25%. The beneficial effects of consuming these vegetables have been observed in cases of CVD, bladder cancer, and oral cancer. Lutein and zeaxanthin are the most common xanthophylls in GLVs, and they are found in kale, spinach, and broccoli. The ratio of lutein to zeaxanthin in GLVs ranges from 12:63, with the highest in kale [13,29,30,31]. A diet high in a variety of foods is important for achieving adequate dietary levels of lutein and zeaxanthin. Moreover, such a diet should include plenty of leafy green vegetables, in keeping with dietary guidelines [31]. Anusha et al. [32] studied 16 GLVs (*Murraya koenigii* (L.) Sprengal, *Trigonella foenum-graecum* L., *Coriandrum sativum* L., *Brassica oleracea* L. var. *italica* Plenck, *Laurus nobilis* L., *Origanum vulgare* L., *O. majorana* L., *Ocimum basilicum* L., *Pandanus amaryllifolius* Roxb., *Salvia officinalis* L., *Thymus vulgaris* L., *Cymbopogon citratus* (DC.) Stapf, *Citrus hystrix* DC. and *Petroselinum crispum* (Mill.) Fuss.) with regard to carotenoid abundance. Of those analyzed, the leaves of *M. koenigii*, *C. sativum*, and *T. foenum-graecum* were rich sources of carotenoids, which may suggest potential antioxidant, anti-inflammatory, and anticancer activities.

### 4.2. Phenolic Compounds and Metabolic Diseases

Phenolic compounds possess a broad spectrum of biochemical activities, such as antioxidant, antimutagenic and anticancer activities, and the ability to modify gene expression. They constitute the largest group of phytochemicals, accounting for most antioxidant activity in plants or plant products. Many studies have shown a direct relationship between plant extracts’ antioxidant activity and phenolic content [33]. Metabolic disorders, including diabetes, obesity and chronic kidney disease, are closely related to oxidative stress and inflammation. Oxidative stress results from an imbalance between the production of reactive oxygen species and the body’s ability to detoxify them, while inflammation involves a chronic immune response contributing to tissue damage and metabolic dysfunction [34]. Managing oxidative stress and inflammation through antioxidant supplementation is an appropriate therapeutic approach. GLVs, rich in phenolic compounds with antioxidant and anti-inflammatory effects, are a good means of natural supplementation (Table 2).

Hydromethanolic extracts of GLVs exhibit anti-inflammatory properties at different levels, and the anti-inflammatory effects may be due to the presence of total polyphenols, flavonoids and carotenoids [37,38]. Arasaretnam et al. [15], investigating the nutritional and mineral composition of selected GLVs, showed total phenolic content ranging from 4.28 ± 1.2 mg GAE/100 g for *Mollugo pentaphylla* L. (Molluginaceae) to 46.07 ± 3.42 mg GAE/100 g for *Delonix elata* (L.) Gamble (Fabaceae). Extracts obtained from common Kenyan GLVs (*Amaranthus dubius*, *Vigna unguiculata*, *Cucurbita maxima*, *Solanum scabrum*) were richer in phenolic antioxidants than wild fruits, and *S. scabrum* had the highest phenolic (92.3 mg GAE/g) and oxygen radical absorbance capacity (ORAC) scores (2675 ± 116 μM trolox equivalent antioxidant capacity (Trolox)/μg) among edible plants [39]. A study by Mulabagal et al. [40] demonstrated that chicoric acid, among other phenolic compounds such as quercetin glucoside, ferulic acid and caffeic acid, isolated from both green and red lettuce, showed 85.6%, 45.6% and 94% inhibition of LPO, COX-1, and COX-2 enzymes, respectively, at a concentration of 50 μM. According to the authors, the variability of phenolic compounds in red and green lettuce, particularly the lack of anthocyanins in green lettuce, may account for the higher antioxidant and anti-inflammatory activity obtained with the red variety. The higher amounts of phenolic compounds, including anthocyanins, present in red lettuce may indicate that it provides better health benefits than green lettuce. Similar results were obtained by Liu et al. [35], showing that leaf lettuce had the highest phenolic content and antioxidant activity, followed by romaine, batavian and butterhead lettuce. Red lettuce varieties contained more phenolic compounds and had more potent antioxidant activity than green lettuce of the same type under the same growth conditions. Bhatt et al. [41] showed that unexplored GLVs such as *Trianthema portulacastrum* L. (Aizoaceae), *Sesbania grandiflora* (L.) Pers. (Fabaceae) and *Plectranthus amboinicus* (Lour.) Spreng. (Lamiaceae) have equivalent or higher phenolic levels and antioxidant properties than commercial GLVs (*Amaranthus blitum* L. Amaranthaceae, *Trigonella foenum-graecum* L. Fabaceae, *Allium cepa* L., Alliaceae), and antioxidant activity correlates significantly with phenolic and flavonoid content. Preliminary in vitro tests of anti-inflammatory activity support the traditional use of these GLVs to alleviate inflammatory diseases.

### 4.3. Antioxidant Vitamins in the Prevention and Treatment of MetS

Vitamins are essential for maintaining healthy body functions. They are crucial for human nutrition because they are organic compounds that humans cannot synthesize independently, necessitating supplementation to prevent metabolic problems [42]. Some vitamins (A, C, E) are well-known antioxidants; however, others, such as vitamin K, vitamin D, niacin, pyridoxine, and riboflavin, are antioxidant in nature and can alleviate oxidative stress, including lipid peroxidation, protein carbonylation, and reduction of advanced glycation end products [42,43]. A rich source of vitamins, including antioxidant compounds, are GLVs (Table 3). For example, riboflavin (vitamin B_2_) dietary sources include spinach, beetroot leaves, asparagus, kale, broccoli, Swiss chard, green beans, turnip leaves, mustard leaves, and cabbage. In contrast, dietary sources of niacin (vitamin B_3_) include cauliflower, broccoli, beet leaves, asparagus, and turnip leaves, among others [43].

There is ample evidence to suggest a potential role for antioxidants in patients with diabetic nephropathy in slowing the progression to advanced stages. Tocotrienols and tocopherols exhibit complex biological activities, including confirmed effects on the vascular and metabolic systems, suggesting a possible role in the prevention and control of cardiometabolic abnormalities, as well as renal abnormalities commonly seen in obesity and diabetes [51]. Parsley (Petroselinum spp.) has a long tradition of use in the treatment of urinary tract diseases, and contemporary in vitro and in vivo studies reveal numerous actions of various parsley preparations, such as diuretic, antidiabetic, hypouricemic, hypoglycemic, hypotensive, antioxidant, anti-inflammatory, and antiplatelet effects [52]. Nouioura et al. [53] demonstrated the protective potential of an ethanolic extract of *Petroselinum crispum* against paracetamol-induced renal, hepatic, and hematological toxicity in rats. The extract could mitigate histopathological damage observed in the liver and kidney. The authors highlight the likelihood that parsley’s antioxidant and free radical scavenging properties play a key role in its potent hepatoprotective and nephroprotective properties. Parsley extract helped prevent proteinuria and low hemoglobin levels, typical side effects of paracetamol use. Therefore, parsley may be promising in treating liver and kidney disorders—especially in cases of proteinuria. The tocopherol complex of *P. crispum* is dominated by α- and γ-tocopherol, with the former being the most abundant tocopherol. Differences in α-tocopherol (the main form of vitamin E) can be significant concerning the antioxidant activity of this plant [48].

### 4.4. Mineral Compounds in Metabolic Problems

Mineral compounds have several important functions essential to the body’s very existence. These include bone calcification, blood clotting, neuromuscular excitability, acid-base balance, fluid balance, and osmotic regulation. Some minerals are integral components of biologically important compounds such as hemoglobin (Fe), thyroxine (I), insulin (Zn) and vitamin B_12_ (Co). Sulphur is present in thiamine, biotin, lipoic acid and coenzyme A. Several minerals participate as enzyme cofactors in metabolism (e.g., Mg, Mn, Cu, Zn, and K), and some are essential components of enzymes (e.g., Co, Mo, and Se) [54,55]. About 25 metals are considered essential in the human diet. Although they comprise only 5% of a typical human diet, they are essential for normal health and function. Macronutrients (Ca, Mg, K, Na, Cl, P, and S) are minerals that adults require in amounts greater than 100 mg/day or account for less than 1% of total body weight. Trace elements (I, Zn, Si, Fe, Mn, Cu, Co, Mo, F, Cr, and B) are usually defined as minerals that adults require in amounts of 1–100 mg/day or account for less than 0.01% of total body weight. Ultra-trace minerals (Al, As, Br, Cd, Ge, Ni, Se, Si, Sn, Pb, Rb, Li, and V) are typically defined as minerals that adults require in amounts less than 1 μg/day [56,57]. Disturbed or unbalanced micronutrient intake adversely affects human health and potentially contributes to the development of metabolic diseases. Hypertension and comorbidities are often characterized by inadequate mineral intake and mineral status disorders [55]. Suliburska et al. [58] estimated the association of mineral status and dietary mineral supply with body mass index (BMI) and serum glucose and lipid levels in patients with newly diagnosed hypertension. The authors showed that Fe, Cu, and Zn levels were associated with metabolic parameters such as BMI and lipid and glucose levels in newly diagnosed hypertensive patients. Serum Zn levels were positively related to triglyceride levels, urinary Fe levels were negatively related to cholesterol levels, and there was an inverse relationship between serum Fe levels and BMI. Vegetables contain essential amounts of macronutrients, which are fundamental to preventing many diseases, including those of metabolic origin. However, due to the increased consumption of processed foods, a large proportion of the population has experienced adverse effects on macronutrient intake [59]. Modern diets should be supplemented with natural sources of minerals, including various plants with edible leaves. The assortment of GLVs has recently expanded considerably to include species that grow wild or have previously been used in other ways (e.g., as edible seeds, tubers or roots) [60,61,62]. GLVs contain numerous minerals, such as Ca, Fe, Cu, P, Zn, Cl, and Na, which are essential for growth and metabolism, the predominant elements being Ca, K, Fe, and Na. Calcium bioavailability levels can vary considerably between plant materials due to different levels of anti-nutrients. In addition to drinking water, milk and dairy products, commonly available GLVs and herbs are good sources of dietary calcium (Table 4). Manivannan et al. [63] report that cabbage, cauliflower and mint leaves contain high total calcium with high bioavailability.

Vegetables provide an alkalizing effect on the acidity produced by other foods, especially those of animal origin [15], with the mineral composition of GLVs being dependent on many factors (genotype, environment, post-harvest conditions, processing). Mineral content profiling of vegetables is important due to possible deficiencies of minerals such as Ca, Fe, and Zn, especially in young children, pregnant women and women of childbearing age. Low intake, poor absorption, excessive Ca loss or a combination of these factors contribute to Ca deficiency diseases such as osteoporosis, hypertension and colorectal cancer. Research by Allen et al. [61] indicates that Ca contained in drumstick tree (*Moringa oleifera* Lam.) leaves does not appear to be associated with low bioavailability complexes such as oxalates. The authors confirmed previous reports that Ca bioavailability in spinach is low and suggest that increased use of *Ipomoea batatas* L. (Lam.) and *M. oleifera* leaves may increase Ca intake in the tropical and warm temperate regions where these plants grow or that these plants may become a valuable export crop.

### 4.5. Anti-Nutritional Substances of GLVs

Edible plants contain numerous substances that affect the human body in different ways. The type and composition of nutritional and anti-nutritional factors depend on the edible plant species. Anti-nutrients prevent the utilization of nutrients or have a detrimental effect on the human body. They can interfere with digestion, absorption, and other steps of nutrient metabolism, thereby reducing their utilization as energy or building blocks [64,65]. Oxalic acid [(COOH)_2_] (OXA) is an anti-nutritional compound that occurs naturally, mainly in plant products; it forms oxalates when combined with salts or minerals. It is present in the cell sap of many GLVs. Various factors affect oxalic acid levels in foods, including species, agricultural traits and consumption practices. Depending on the plant species, oxalates can occur as insoluble salts of calcium, magnesium, and iron and soluble salts of potassium, sodium, ammonium and lithium or as a combination of the two forms. The ascorbic acid metabolism endogenously synthesizes oxalates, and glyoxalates are the end product of alanine, glycine, and serine metabolism [64,66]. The anti-nutritional effect of oxalates mainly concerns the soluble forms. Insoluble oxalates are excreted, while soluble oxalates affect the human body by forming a strong chelate with calcium and other minerals, making the complex unavailable for absorption and assimilation. Insoluble calcium oxalate in crystalline form is stored in the kidneys, causing a serious health problem (kidney stones). This process reduces the availability of calcium ions in the blood plasma, disrupting calcium-phosphate metabolism. The amount of oxalate is high in leaves, followed by seeds and stems; high amounts of oxalic acid are contained in, among others, spinach, rhubarb and beetroot leaves. The bioavailability of oxalates from various dietary sources ranges from 2–15% (1% for rhubarb and spinach and 22% for tea). However, it is not only the oxalic acid content of the products that is important, but also its ratio to calcium content (Oxa/Ca), which should be less than 1.0. For example, parsley, on average, contains 140–200 mg/100 g oxalic acid, but at the same time, it contains 180–290 mg/100 Ca; therefore, the Oxa/Ca ratio is favorable at only 0.32 [64]. It should be mentioned that GLVs generally contain fewer anti-nutrients than the recommended daily intake for humans, which is defined as 50 mg/100 g (Table 5).

## 5. Dietary GLVs and Metabolic Protection

### 5.1. GLV-Enriched Diet and Diabetes Prevention

Diabetes is a chronic disease characterized by abnormalities in the secretion or action of insulin, or sometimes both. The disease has become a serious and growing global health burden. According to the WHO, it affects 9.0% of the world’s adult population. It is estimated that the number of people with diabetes could increase to 592 million by 2035. The progression of the disease over time is associated with increased disability and mortality [68,69,70]. The incidence of T2DM is related to diet. Consumption of GLVs minimizes the risk of diabetes. Some GLVs (e.g., *Amaranthus species*, *Brassica chinensis*, *Chenopodium album*, *Portulaca oleracea*) have shown differential antidiabetic effects in vitro and in vivo [70]. Patients who consumed higher amounts of certain vegetables (including GLVs) and fruits showed lower fasting blood sugar levels [71]. Chen et al. [72] report that intake of GLVs was not significantly associated with T2DM risk in the Asian population. However, there is evidence that higher intake of GLVs reduces the risk of T2DM [73,74,75]. Wang et al. [73] found no significant association between total vegetable intake and the risk of T2DM. However, they found that increasing the intake of GLVs, yellow vegetables, or cruciferous vegetables may help reduce the risk of T2DM. The authors also found that high intake of plant fiber reduced the risk of T2DM. Yen et al. [74] conducted a study among people with T2DM and their family members. A fiber-rich diet and GLVs showed an inverse relationship with lower T2DM risk and better glycemic control. The authors emphasize that adding vegetables to the diet reduces overall energy density, increasing the amount of food that can be consumed at a given calorie level. At the same time, adding vegetables reduces calorie intake, increases feelings of satiety, and reduces hunger. Similarly, a study by Asekenye et al. [75] reveals that when hyperglycemic patients include GLVs in their diet (in the recommended amount), prepared using methods that preserve and/or increase nutrient and phytonutrient content, they can control and prevent high blood glucose levels. The mechanisms that may lead to lower blood sugar levels are related to the presence of fiber, polyphenols, and other antioxidants. Fiber consumption prevents spikes in blood sugar levels by slowing digestion and reducing the rate of glucose absorption into the bloodstream. Polyphenols and other antioxidants have a protective effect, reducing the effects of free radical damage, as well as anti-inflammatory properties. Polyphenols may also interfere with glucose absorption and carbohydrate catabolism [71].

### 5.2. GLVs as an Active Protective Complex in CVD

Epidemiological studies have shown that vegetable intake is inversely related to CVD risk. Many vegetables, such as tomatoes, onions, celery, broccoli, lettuce, and asparagus, show great potential for preventing and treating CVD due to the presence of vitamins, minerals, dietary fiber, polyphenols and other bioactive compounds. GLVs in the diet significantly reduce the incidence of CVD (15.8%) [76]. GLVs increase antioxidant capacity due to the minerals, vitamins, and phytochemicals they contain, which protect against oxidative stress and play an important role in the pathogenesis of CVD. In traditional diets with a high intake of GLVs (Mediterranean and Japanese diets), CVD rates are lower, and life expectancy is longer. In addition, the incidence of coronary heart disease tends to be lower in people who consume more GLVs. GLVs such as arugula (*Eruca sativa* (L.) Cav.), spinach (*Spinacia oleracea* L.) and lettuce (*L. sativa* L.) lower blood pressure, inhibit platelet aggregation and improve endothelial dysfunction due to their high content of inorganic nitrates. These vegetables protect against cancer, especially cancer of the digestive system. Spinach has a protective effect against cancer by reducing oxidative stress in the body due to the vitamins A, C, and E, carotenes such as β-carotene and lutein, and flavonoids it contains [13].

### 5.3. GLVs Against Obesity

Purslane (*Portulaca oleracea* L.) and chard (*Beta vulgaris* L. var. *cicla* L.) are GLVs that have functional effects against MetS and obesity. Purslane has functional effects due to free oxalic acids, alkaloids, omega-3 fatty acids, coumarins, flavonoids, cardiac glycosides, anthraquinones, and other bioactive compounds. In contrast, chard shows its effects mainly through phospholipids, glycolipids, fatty acids (palmitic, stearic, oleic, and linoleic), folate, ascorbic acid and pectin [13]. Jang et al. [77] showed that *P. oleracea* extract regulates hepatic cholesterol metabolism in rats fed a cholesterol-rich diet by modulating the liver’s AMP-activated protein kinase (AMPK) activation and microRNA (miR)-33/34a expression. The results suggest that purslane extract improves serum, liver, and fecal lipid profiles and positively alters the expression of genes involved in cholesterol metabolism in the liver in rats fed a high-cholesterol diet. The authors suggest that purslane extract may be useful as a potential functional food to improve CVD. In addition, purslane extract appears to be a safe adjunctive treatment for T2DM, significantly lowering systolic blood pressure. Studies evaluating the effect of purslane extract use on anthropometric and biochemical changes in patients with T2DM have found reductions in glycated hemoglobin (HbA1c), triglycerides (TG), total cholesterol (TC), low density lipoprotein (LDL), fasting blood glucose (FBG), post-meal blood glucose, body weight, and body mass index (BMI), and significant increases in high-density lipoproteins (HDL) [78,79]. In addition, consumption of purslane extract significantly increases glucagon-like peptide-1, which positively affects beta-cell proliferation and insulin secretion in people with T2DM [80].

In conclusion, the nature of MetS as a multidimensional condition suggests that the best therapeutic approach is to act simultaneously in a holistic and personalized manner, addressing all components of MetS: obesity, fat remodeling, dyslipidemia, diabetes, insulin resistance, and hypertension [81]. Consumption of GLVs, which are nutrient-rich and health-promoting, is associated with human health and reduced risk of various diseases, including MetS. GLVs promote a healthy state and alleviate disease states. Their bioactive components (polyphenols, carotenoids, tocopherols, L-ascorbic acid, and minerals) possess antioxidant, anti-inflammatory, and other biological properties. Their role in the prevention of MetS is related to the fact that metabolic diseases are exacerbated by inflammation and oxidative stress. GLVs are a source of health-promoting phytochemicals and can be used by people of all ages. They are useful in alleviating and combating many diseases caused by deficiency. GLVs are a source of fiber and bioactive compounds such as polyphenols, carotenoids, phytosterols, vitamins and minerals. Regular consumption of GLVs is associated with reduced CVD risk factors, such as reduced oxidative stress, regulated post-meal blood glucose levels, and modulated lipid metabolism. In addition, the dietary fiber in GLVs inhibits the activity of carbohydrate-digesting enzymes and slows glucose absorption, leading to lower post-meal glucose levels. GLVs contain phytosterols, compounds structurally similar to cholesterol, which can lower blood lipid levels in rats and humans. In addition, GLVs contain nitrates, which can be converted to nitric oxide via a non-enzymatic synthesis pathway, promoting vasodilation, improving endothelial function, and lowering blood pressure, thereby reducing CVD risk [10].

The high nutritional and dietary value of GLVs contributed to the search for ways to enrich them with substances important for health. Biofortification has proven to be such a method—a significant innovation in the horticultural sector. Biofortification of GLVs with selenium is an example of enriching these plants with a trace element that has a beneficial effect on human health [82]. Considering the recommended daily allowance (RDA) of iodine, consumption of one serving of *Brassica rapa* L. subsp. *narinosa* microgreens biofortified with an 8 μM iodine solution can cover 100.19% and 37.57% of the daily requirement for adults and children, respectively [83]. The data presented indicate the significant potential of biofortification of GLVs in the production of effective and sustainable food.

## 6. Conclusions

MetS is an extensive group of conditions that are associated with specific disturbances in human metabolic processes. The global prevalence of these diseases has increased over the past two decades. Prevalence rates have increased for all MetS components between 2000 and 2019, with the most significant increases in countries with high socio-demographic indicators [84]. GLVs, a unique, highly diverse group of vegetables, recently expanded to include valuable wild and cultivated species classified in separate groups (root vegetables, legumes, and others), which provide excellent support for human metabolism and protection against many diseases. The phytochemical and nutritional profiles of GLVs can be an important part of the daily diet, helping to protect the body against various MetS and diseases resulting from a violation of normal metabolism. Over 350,000 plant species have been described, and thousands of newly identified species are added to the global list annually. Of these known plants, more than 7000 documented species and possibly as many as 30,000 plants can be considered edible for humans, with at least 7000 being cultivated for food and agriculture [85]. Among those listed may be GLVs not yet described, which should be investigated for their nutritional and health-promoting value, safety of use, and usefulness against MetS. It is advisable to isolate and identify the bioactive components and investigate the underlying mechanisms of action and related clinical trials.

## Figures and Tables

**Table 1:** Carotenoid content in edible parts of some green leafy vegetables [27,28].

Green Leafy Vegetables	α-Carotene	β-Carotene	Lutein + Zeaxanthin
	mg/100 g Edible Parts		
Cabbage (*Brassica oleracea* L. var. *capitata* L. f. alba)	0–0.002	0.01–0.41	0.084–0.45
Cabbage (*Brassica oleracea* L. var. *capitata* L. f. rubra)	tr	0.015–0.05	0.026–0.15
Brussels sprouts (*Brassica oleracea* L. var. *gemmifera* DC.)	0.004–0.011	0.43–1.02	0.92–1.59
Kale (*Brassica oleracea* L. var. *sabellica* L.)	0–0.15	2.84–43.80	3.04–39.55
Spinach (*Spinacia oleracea* L.)	0–0.09	3.25–5.60	4.40–11.94
Lettuce (*Lactuca sativa* L.)	0.04	0.98–1.55	1.35–2.92
Leek (*Allium ampeloprasum* L.)	-	1.00–3.19	1.90–3.68
Lamb’s lettuce (*Valerianella locusta* (L.) Laterr)	0.08	3.22	9.65
Parsley (*Petroselinum crispum* (Mill.) Fuss)	0.17	5.50	14.12

**Table 2:** Phenolic content and antioxidant activity of some green leafy vegetables) [22,35,36].

Green Leafy Vegetables	mg GAE/g	% DPPH *
*Lactuca sativa* L. green cv. (Asteraceae)	21.0 ± 5.1–34.7 ± 3.6	74.4 ± 6.5–84.2 ± 1.0
*Lactuca sativa* L. red cv. (Asteraceae) *Anethum graveolens* L. (Apiaceae)	55.2 ± 4.4–73.9 ± 19.8 315.79 ± 1.38	81.2 ± 4.0–82.6 ± 1.8 114.5 ± 1.27
*Hibiscus sabdariffa* L. (Malvaceae) *Corchorus oletorius* L. (Malvaceae) *Cucumis melo* L. (Cucurbitaceae) *Momordica charantia* L. (Cucurbitaceae)	212.49 ± 0.27 305.04 ± 0.51 198.26 ± 0.35 361.80 ± 0.73	26.23 ± 0.28 36.14 ± 0.39 16.17 ± 0.10 52.65 ± 0.43

* Total antioxidant capacity expressed using the DPPH radical reduction method.

**Table 3:** The content of antioxidant vitamins in some selected green leafy vegetables [44,45,46,47,48,49,50].

Green Leafy Vegetables	L-Ascorbic Acid mg/100 g FW	Total Tocopherols μg/g FW
*Petroselinum crispum* (Mill.) Fuss. (Apiaceae)	133.0	19.8 ± 0.6–31 ± 2
*Anethum graveolens* L. (Apiaceae)	85.0	68.65
*Lactuca sativa* L. (Asteraceae) *Brassica oleracea* L. (Brassicaceae)	24.0–30.2 51.0	274.7–526.5 0.03–0.82

**Table 4:** Calcium content of selected green leafy vegetable species [31,47].

Green Leafy Vegetables	Ca
*Spinacia oleracea* L. (Chenopodiaceae)	1036 mg/100 g of product
*Petroselinum crispum* (Mill.) Fuss. (Apiaceae)	138 mg/100 g dry weight (DW)
*Anethum graveolens* L. (Apiaceae)	208 mg/100 g DW
*Lactuca sativa* L. (Asteraceae)	36 mg/100 g DW
*Brassica oleracea* L. (Brassicaceae)	47 mg/100 g DW

**Table 5:** Anti-nutrient content of some **green leafy vegetables** [67].

Species	Oxalate	Tannin	Phytate
	mg/100 g DW		
Cabbage (*Brassica oleracea* L.)	0.88 ± 0.06	261.76 ± 0.23	130.88 ± 0.11
Swiss chard (*Beta vulgaris* L. var. *cicla* L.)	3.56 ± 0.09	531.97 ± 0.01	163.31 ± 0.50
Celery (*Apium graveolens* L.)	3.18 ± 0.03	327.62 ± 0.11	265.99 ± 0.00

## Data Availability

No new data were created or analyzed in this study.
